# Association between sodium–glucose cotransporter-2 inhibitors and arrhythmic outcomes in patients with diabetes and pre-existing atrial fibrillation

**DOI:** 10.1093/europace/euae054

**Published:** 2024-03-14

**Authors:** Akash Fichadiya, Amity Quinn, Flora Au, Dennis Campbell, Darren Lau, Paul Ronksley, Reed Beall, David J T Campbell, Stephen B Wilton, Derek S Chew

**Affiliations:** Libin Cardiovascular Institute, Department of Cardiac Sciences, Cumming School of Medicine, University of Calgary, 3330 Hospital Drive NW, T2N 4N1, Calgary, AB, Canada; Department of Community Health Sciences, Cumming School of Medicine, University of Calgary, 3280 Hospital Drive NW, T2N 4Z6 Calgary, AB, Canada; O’Brien Institute for Public Health, Cumming School of Medicine, University of Calgary, 3280 Hospital Drive NW, T2N 4Z6 Calgary, AB, Canada; Department of Community Health Sciences, Cumming School of Medicine, University of Calgary, 3280 Hospital Drive NW, T2N 4Z6 Calgary, AB, Canada; Department of Medicine, University of Alberta, 13-103 Clinical Sciences Building, 11350 - 83 Avenue NW, T6G 2G3 Edmonton, AB, Canada; Department of Medicine, University of Alberta, 13-103 Clinical Sciences Building, 11350 - 83 Avenue NW, T6G 2G3 Edmonton, AB, Canada; Department of Community Health Sciences, Cumming School of Medicine, University of Calgary, 3280 Hospital Drive NW, T2N 4Z6 Calgary, AB, Canada; O’Brien Institute for Public Health, Cumming School of Medicine, University of Calgary, 3280 Hospital Drive NW, T2N 4Z6 Calgary, AB, Canada; Department of Community Health Sciences, Cumming School of Medicine, University of Calgary, 3280 Hospital Drive NW, T2N 4Z6 Calgary, AB, Canada; O’Brien Institute for Public Health, Cumming School of Medicine, University of Calgary, 3280 Hospital Drive NW, T2N 4Z6 Calgary, AB, Canada; Libin Cardiovascular Institute, Department of Cardiac Sciences, Cumming School of Medicine, University of Calgary, 3330 Hospital Drive NW, T2N 4N1, Calgary, AB, Canada; Department of Community Health Sciences, Cumming School of Medicine, University of Calgary, 3280 Hospital Drive NW, T2N 4Z6 Calgary, AB, Canada; O’Brien Institute for Public Health, Cumming School of Medicine, University of Calgary, 3280 Hospital Drive NW, T2N 4Z6 Calgary, AB, Canada; Department of Medicine, Cumming School of Medicine, University of Calgary, 3330 Hospital Drive NW, T2N 4N1 Calgary, AB, Canada; Libin Cardiovascular Institute, Department of Cardiac Sciences, Cumming School of Medicine, University of Calgary, 3330 Hospital Drive NW, T2N 4N1, Calgary, AB, Canada; Department of Community Health Sciences, Cumming School of Medicine, University of Calgary, 3280 Hospital Drive NW, T2N 4Z6 Calgary, AB, Canada; Department of Medicine, Cumming School of Medicine, University of Calgary, 3330 Hospital Drive NW, T2N 4N1 Calgary, AB, Canada; Libin Cardiovascular Institute, Department of Cardiac Sciences, Cumming School of Medicine, University of Calgary, 3330 Hospital Drive NW, T2N 4N1, Calgary, AB, Canada; Department of Community Health Sciences, Cumming School of Medicine, University of Calgary, 3280 Hospital Drive NW, T2N 4Z6 Calgary, AB, Canada; O’Brien Institute for Public Health, Cumming School of Medicine, University of Calgary, 3280 Hospital Drive NW, T2N 4Z6 Calgary, AB, Canada; Department of Medicine, Cumming School of Medicine, University of Calgary, 3330 Hospital Drive NW, T2N 4N1 Calgary, AB, Canada

**Keywords:** SGLT2 inhibitor, DPP4 inhibitor, Atrial fibrillation, Type II diabetes, Hospitalization, Heart failure

## Abstract

**Aims:**

Prior studies suggest that sodium–glucose cotransporter-2 inhibitors (SGLT2is) may decrease the incidence of atrial fibrillation (AF). However, it is unknown whether SGLT2i can attenuate the disease course of AF among patients with pre-existing AF and Type II diabetes mellitus (DM). In this study, our objective was to examine the association between SGLT2i prescription and arrhythmic outcomes among patients with DM and pre-existing AF.

**Methods and results:**

We conducted a population-based cohort study of adults with DM and AF between 2014 and 2019. Using a prevalent new-user design, individuals prescribed SGLT2i were matched 1:1 to those prescribed dipeptidyl peptidase-4 inhibitors (DPP4is) based on time-conditional propensity scores. The primary endpoint was a composite of AF-related healthcare utilization (i.e. hospitalization, emergency department visits, electrical cardioversion, or catheter ablation). Secondary outcome measures included all-cause mortality, heart failure (HF) hospitalization, and ischaemic stroke or transient ischaemic attack (TIA). Cox proportional hazard models were used to examine the association of SGLT2i with the study endpoint. Among 2242 patients with DM and AF followed for an average of 3.0 years, the primary endpoint occurred in 8.7% (*n* = 97) of patients in the SGLT2i group vs. 10.0% (*n* = 112) of patients in the DPP4i group [adjusted hazard ratio 0.73 (95% confidence interval 0.55–0.96; *P* = 0.03)]. Sodium–glucose cotransporter-2 inhibitors were associated with significant reductions in all-cause mortality and HF hospitalization, but there was no difference in the risk of ischaemic stroke/TIA.

**Conclusion:**

Among patients with DM and pre-existing AF, SGLT2is are associated with decreased AF-related health resource utilization and improved arrhythmic outcomes compared with DPP4is.

## Graphical Abstract

**Graphical Abstract:**
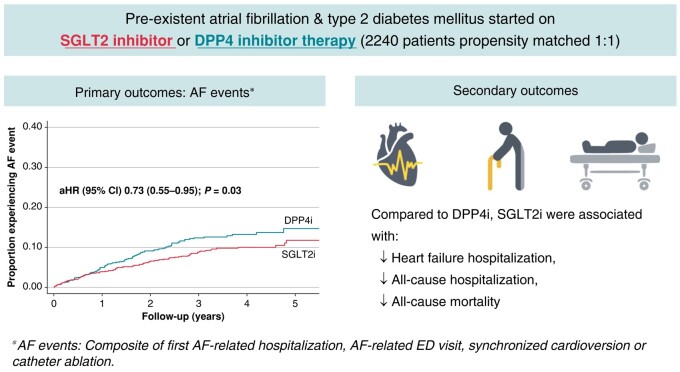

## Introduction

Sodium–glucose cotransporter 2 inhibitors (SGLT2is) were originally developed as glucose-lowering agents for individuals with Type II diabetes mellitus (DM).^1^ More recently, the role of SGLT2i has expanded to include patients with atherosclerotic cardiovascular disease, heart failure (HF), and chronic kidney disease (CKD). Among these patient populations, randomized trials have demonstrated that SGLT2i decreases the frequency of HF-related hospitalizations, reduces the risk of cardiovascular and all-cause death, improves health-related quality of life, and attenuates the progression of kidney disease.^2,3^

Furthermore, there is emerging evidence that SGLT2i may affect arrhythmia-related outcomes. For example, *post hoc* analyses of the landmark SGLT2i trials suggested a decreased incidence of atrial fibrillation (AF) and atrial flutter (AFL) among patients treated with SGLT2i compared with placebo.^4^ In a meta-analysis of 31 trials comprised of over 75 000 participants, SGLT2is were associated with a lower risk of serious AF events compared with placebo [risk ratio 0.75; 95% confidence interval (CI) 0.66–0.86].^4^ However, these prior studies hadsolely examined the relationship between SGLT2i and the development of new-onset AF, with limited research among patients with pre-existing AF.

Notably, the effects of SGLT2i on the natural history of pre-existing or established AF are unknown. It is plausible that SGLT2i may attenuate the progression of AF arrhythmic burden and the corresponding frequency of AF-related healthcare encounters. Although the exact non-glycaemic benefits of SGLT2i are incompletely understood, effects on neurohumoral activation and intra-cellular ion homeostasis may drive favourable metabolic improvement and structural remodelling of the heart (both atrial and ventricular), which may improve the natural history of AF.^5^

The objective of the current population-based cohort study was to examine the association between SGLT2i and arrhythmic outcomes, including AF-related healthcare utilization, in patients with DM and pre-existing AF.

## Methods

The analysis was approved by the Conjoint Health Research Ethics Board at the University of Calgary and conducted in accordance with the Declaration of Helsinki. Individual written informed consent was waived owing to the fully de-identified structure of the dataset.

### Study design and setting

We conducted a retrospective, population-based cohort study of adults with DM and AF between 1 June 2014 and 31 March 2019, in Alberta, Canada, a province of ∼4.4 million people served by a single healthcare system,^6^ using a prevalent new-user design (see the Propensity score matching section).^7^ All Alberta residents are eligible for public health insurance, and >99% participate in the government-sponsored insurance plan, which covers physician visits and hospital-based care—but does not universally cover pharmaceuticals.^8^ Each resident is assigned a personal health number, which acts as a unique lifetime identifier that enables the linkage of administrative health data maintained by Alberta Health.^9^

### Data sources

We accessed de-identified data from the Interdisciplinary Chronic Disease Collaboration Data Repository,^10^ which contains linked administrative health, pharmacy, and laboratory patient-level data and includes demographic characteristics, vital statistics, physician claims, hospital admissions, emergency department and ambulatory care visits, laboratory results, and dispensed prescription data for the entire adult (≥18 years) population.

### Study cohort

We identified a population-based cohort of adults (≥18 years) with both DM and AF as of 31 March 2017, leaving at least 2 years for outcome ascertainment. Eligible patients were identified if they had one hospitalization or two physician claims at least 30 days apart for both diabetes (excluding gestational diabetes) and AF, using previously validated administrative coding algorithms.^11^ Patients without an indication for an SGLT2i were excluded: (i) Stages G4 and G5 CKD (estimated glomerular filtration rate [eGFR] < 30 mL/min/1.73 m^2^), (ii) prior lower limb amputation, and (iii) HbA1C < 7.5 who were taking only metformin (defined as taking metformin with no other agents ±365 days from the study start date). We also excluded individuals with Type I DM using a previously validated method based on the sole prescription of insulin monotherapy.^12^

The study cohort comprised adults with DM and AF who were dispensed SGLT2i (canagliflozin, dapagliflozin, or empagliflozin) or dipeptidyl peptidase-4 inhibitor (DPP4i) (alogliptin, linagliptin, saxagliptin, or sitagliptin) that was available in Canada during the study period. Patients were classified into one of two mutually exclusive groups: (i) patients using SGLT2i alone or in combination with other non-DPP4i diabetic drugs or (ii) patients using DPP4i alone or in combination with other non-SGLT2i drugs. Patients simultaneously starting SGLT2i and DPP4i on the same date were excluded. Dipeptidyl peptidase-4 inhibitor exerts hypoglycaemic effects by increasing incretin levels [glucagon-like peptide-1 (GLP-1) and gastric inhibitory peptide] and inhibits glucagon release, which, in turn, increase insulin secretion, decrease gastric emptying, and decrease blood glucose levels. Dipeptidyl peptidase-4 inhibitors were chosen as an active comparator agent to reduce the risk of immortal time bias in the study design,^13^ and since they were similarly considered a second-line or third-line agent for glucose-lowering during the study period, they demonstrated cardiovascular safety and were thought to have neutral cardiovascular effects.^14–16^

### Propensity score matching

**Figure 1:**
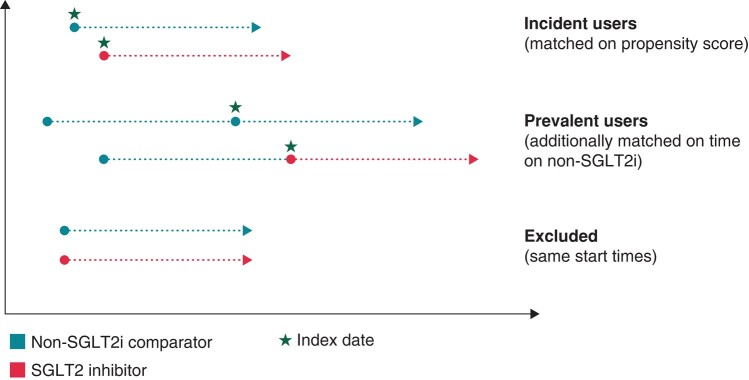
A schematic of a prevalent new-user design. Prevalent new users of SGLT2i and incident new users of SGLT2i (i.e. people who had not previously used DPP4i) were matched to DPP4i. The index date of outcome ascertainment was defined by the date when the SGLT2i was dispensed or the corresponding date when the DPP4i was dispensed in the matched exposure. Patients were followed up until death, emigration out of province, termination of registration with Alberta Health, or until end of the study period (31 March 2019). DPP4i, dipeptidyl peptidase-4 inhibitor; SGLT2i, sodium–glucose cotransporter-2 inhibitor.

This study used a prevalent new-user cohort design described in previous Canadian pharmaco-epidemiology studies of SGLT2i outcomes.^7,17,18^ In brief, we included both prevalent new users of SGLT2i (i.e. people who previously used DPP4i) and incident new users of SGLT2i (i.e. people who had not previously used DPP4i) matched to DPP4i (*Figure 1*). The index date of outcome ascertainment was defined by the date when SGLT2i was dispensed or the corresponding date when DPP4i was dispensed in the matched exposure. Patients were followed up until death, emigration out of province, termination of registration with Alberta Health, or until the end of the study period (31 March 2019).

We first created exposure sets of potential matches defined by the level of diabetes therapy based on the number of diabetic medications, duration of DPP4i treatment for prevalent new users, and calendar time (DPP4i prescription within 180 days of the SGLT2i initiation). We matched incident SGLT2i users to incident DPP4i users who were initiated treatment in the same period, whereas we matched patients switching from a DPP4i to an SGLT2i (prevalent new users) to patients treated with DPP4i for a similar duration in their exposure sets.

Propensity scores were then constructed separately for incident and prevalent new users to estimate the propensity of receiving an SGLT2i vs. a DPP4i. We used logistic regression to model the association of the following covariates and receiving an SGLT2i vs. a DPP4i: age, sex, diabetes duration, AF duration, comorbidities (myocardial infarction, cirrhosis, CKD, HF, hypertension, thyroid disease, peripheral artery disease, stroke, cancer, and peripheral vascular disease), anti-hyperglycaemic medications (insulin, GLP-1 receptor agonists, alpha-glucosidase, meglitinides, metformin, sulfonylureas, thiazolidinediones), AF medications (oral anticoagulants, beta-blockers, calcium channel blockers, and anti-arrhythmics), CHADS_2_ score, and health services utilization (number of diabetes medications, number of hospital visits, and number of outpatient consultations). Sodium–glucose cotransporter 2 inhibitor users were matched 1:1 using the nearest neighbour method with replacement to DPP4i users and using a caliper width of 0.2 SD of the log of the propensity score.

### Covariates

Baseline comorbidities were ascertained from administrative data based on previously validated algorithms over a 2-year look-back period.^11,19^ We used the Pampalon Deprivation Index as a comprehensive indicator of socioeconomic status, which is a small-area–based composite index derived from census data that include employment status, income, education, marital status, single-parent status, or living alone.^20,21^ The material index reflects deprivation of wealth, goods, and conveniences, and the social index reflects deprivation of relationships among individuals in the family, the workplace, and the community. Each index stratifies each dissemination area (i.e. smallest standard census area) into quintiles, from 1 (least deprived) to 5 (most deprived), and is assigned to individuals in the cohort based on postal code.^22^

Medications were identified using Anatomical Therapeutic Chemical codes and included rate control medications for AF (i.e. beta-blockers excluding sotalol, non-dihydropyridine calcium channel blockers, and digoxin), anti-arrhythmic medications (i.e. Vaughan Williams Class IA and IC agents, amiodarone, and sotalol), anticoagulants (i.e. warfarin, rivaroxaban, apixaban, dabigatran, and edoxaban), and anti-hyperglycaemic medications (i.e. metformin, insulin, sulfonylureas, alpha-glucosidase inhibitors, meglitinides, GLP-1 agonists, SGLT2i, and DPP4i).

### Outcomes

The primary outcome was an ‘AF event’ or a clinically relevant AF-related medical resource encounter. Atrial fibrillation events were defined as the first occurrence of an AF-related hospitalization, AF-related emergency department visit, synchronized electrical cardioversion, or catheter ablation. Secondary outcomes included all-cause mortality, all-cause hospitalization, HF hospitalization, and ischaemic stroke or transient ischaemic attack (TIA). Administrative definitions and data sources used to define each outcome are listed in Supplementary material online, *Table S1*.

### Statistical analysis

Descriptive statistics were used to summarize covariates by the propensity-matched treatment group. Continuous variables were reported as means and standard deviations, and categorical variables were reported as proportions. The balance of covariates was assessed based on standardized differences with a threshold of 10%.

All analyses were conducted using an intention-to-treat principle. Kaplan–Meier curves were used to visualize the cumulative incidence of primary and secondary outcomes over time, and log-rank tests were used to compare the survival distribution by treatment groups. The relationship between treatment (SGLT2i vs. DPP4i comparator) and study outcomes was assessed using Cox regression models without competing risks to calculate hazard ratios (HRs) and 95% confidence intervals (95% CI). Cox proportional hazard modelling was chosen to facilitate clinical interpretability of HR.^23^ Adjusted multivariable Cox proportional hazard regression was used to balance and control for covariates with a standardized difference >10% following matching.^24^ Time zero was set to the date of the first SGLT2i prescription or the date of the DPP4i prescription in the corresponding time-conditional exposure sets. We tested the proportional hazards assumption for the Cox proportional hazards model by examining the Schoenfeld residuals and visual assessment of log–log plots. The proportional hazards assumption was met across models. The analyses were repeated within pre-defined subgroups including female sex, HF, CKD, and the baseline use of anti-arrhythmic medications.

As a secondary analysis, Fine–Gray models were used for non-fatal outcomes to account for the competing risk of death.^25^ In addition to the time-to-event analyses, we conducted recurrent event analyses of the primary study endpoint using several approaches including (i) the Andersen and Gill model; (ii) the Prentice, Williams, and Petersen total time model; and (iii) a random effects approach using a frailty model.^26–28^

To evaluate the potential for residual confounding, falsification analysis was conducted by ascertaining the relationship between the treatment group and falsification endpoints that *a priori* would not be expected to be associated with the effects of treatment. For this study, we considered incident rheumatoid arthritis, chronic obstructive pulmonary disease, and lymphoma. Statistical analyses were performed with SAS, version 9.4 (SAS Institute Inc.), and Stata/IC, version 14.2 (StataCorp LP).

## Results

### Baseline characteristics

**Figure 2:**
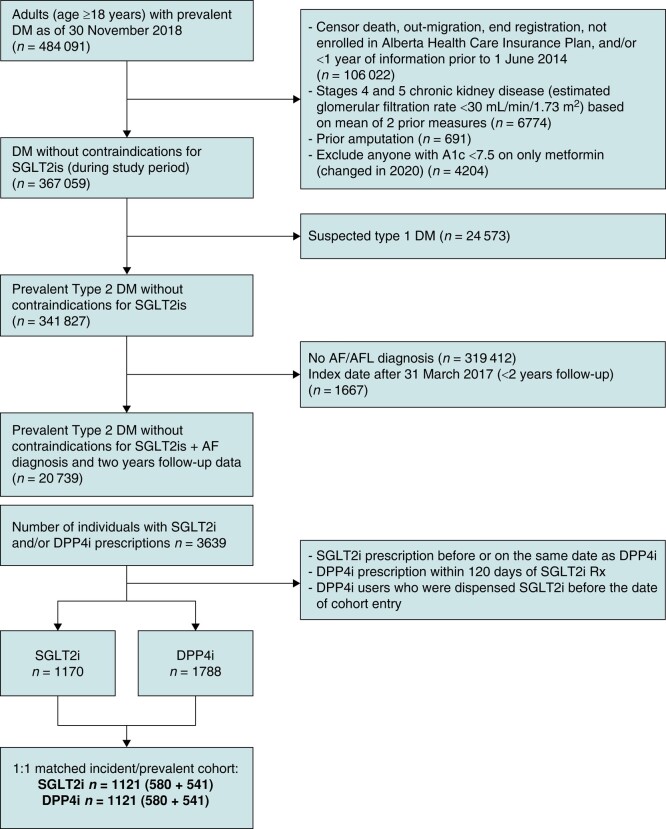
A study flow diagram. There were 20 739 patients with concomitant DM and AF without contraindications to SGLT2i. Following 1:1 matching with replacement, the final propensity-matched cohort included a total of 2242 patients (1121 in each group). AF, atrial fibrillation; AFL, atrial flutter; DM, diabetes mellitus; DPP4i, dipeptidyl peptidase-4 inhibitor; SGLT2i, sodium–glucose cotransporter-2 inhibitor.

Among 20 739 patients with concomitant DM and AF and without contraindications to SGLT2i, there were 1170 patients prescribed an SGLT2i and 1788 prescribed a DPP4i during our accrual period (*Figure 2*). Following 1:1 matching with replacement, the final propensity-matched cohort included a total of 2242 patients (1121 in each group) followed for a median of 3.0 years (inter-quartile range 2.1–4.1).

The mean age of the overall matched cohort was 66 years, 26% were female, and the mean CHADS_2_ score was 2.3 (*Table 1*) with 53% on anticoagulation for stroke prevention. The most common comorbidities in both groups included hypertension (93%), CKD (83%), and HF (40%). The majority of baseline characteristics between the SGLT2i and DPP4i groups was balanced (i.e. standardized difference <10%) after propensity score matching. Notably, the mean duration of AF (i.e. time from AF diagnosis to the initiation of SGLT2i or DPP4i) was similar between groups (6.6 ± 4.8 years in the SGLT2i group vs. 6.9 ± 4.8 years in the DPP4i group; standardized difference 5.7%). The proportion of patients on anti-arrhythmic drugs at baseline were also similar between groups.

**Table 1:** Baseline characteristics of users of SGLT2i and matched users of DPP4i

Characteristics	SGLT2 inhibitor (*N* = 1121)	DPP4 inhibitor (*N* = 1121)	Standardized difference (%)
Age, mean (SD), years	64.8 (10.2)	68.0 (10.5)	30.9
Age group, *n* (%)			
<65	548 (48.9)	428 (38.2)	21.7
≥65	572 (51.1)	693 (61.8)	
Female sex, *n* (%)	277 (24.7)	304 (27.1)	5.5
Urban/rural, *n* (%)			
Urban	923 (82.3)	954 (85.1)	7.0
Rural	193 (17.2)	165 (14.7)	
Missing	5 (0.5)	2 (0.2)	
Pampalon material deprivation, *n* (%)			
5 (most deprived)	91 (23.1)	94 (23.9)	1.8
4	102 (25.9)	63 (16.0)	19.8
3	69 (17.5)	84 (21.3)	9.6
2	58 (14.7)	53 (13.5)	3.6
1 (less deprived)	57 (14.5)	63 (16.0)	4.2
Not defined	17 (4.3)	30 (7.6)	14.0
Pampalon social deprivation, *n* (%)			
5 (most deprived)	287 (25.6)	326 (29.2)	7.8
4	275 (24.5)	250 (22.3)	5.3
3	198 (17.7)	212 (18.9)	3.3
2	143 (12.8)	133 (11.9)	2.7
1 (less deprived)	169 (15.1)	123 (11.0)	12.2
Not defined	49 (4.4)	77 (6.9)	10.8
Comorbidities, *n* (%)			
0–1	–	–	–
2	10 (0.9)	18 (1.6)	6.4
≥3	1111 (99.1)	1103 (98.4)	6.4
Hypertension, *n* (%)	1030 (91.9)	1055 (94.1)	8.7
Heart failure, *n* (%)	393 (35.1)	494 (44.1)	18.5
Acute myocardial infarction, *n* (%)	158 (14.1)	180 (16.1)	5.5
Peripheral artery disease, *n* (%)	42 (3.7)	77 (6.9)	14.0
Ischaemic stroke/TIA, *n* (%)	204 (18.2)	262 (23.4)	12.8
Chronic kidney disease, *n* (%)	912 (81.4)	941 (83.9)	6.8
Cancer, *n* (%)	54 (4.8)	72 (6.4)	7.0
Pulmonary disease, *n* (%)	1 (0.1)	3 (0.3)	4.2
Thyroid, *n* (%)	149 (13.3)	176 (15.7)	6.8
Cirrhosis, *n* (%)	9 (0.8)	15 (1.3)	5.2
AF characteristics			
CHADS_2_ score, mean (SD)	2.2 (1.2)	2.4 (1.3)	15.9
Years since AF diagnosis, mean (SD)	6.6 (4.8)	6.9 (4.8)	5.7
Prior catheter ablation, *n* (%)	4 (0.6)	4 (0.6)	0.0
Diabetes characteristics			
Years since DM diagnosis, mean (SD)	9.6 (6.1)	10.2 (6.2)	5.6
Number of different classes of antiglycaemic agents, *n* (%)			
0–1	136 (12.1)	132 (11.8)	1.1
2–3	671 (59.9)	661 (59.0)	1.8
4+	314 (28.0)	328 (29.3)	2.8
Medications in prior year, *n* (%)			
Oral anticoagulation	570 (50.8)	625 (55.8)	9.8
Beta-blocker	698 (62.3)	734 (65.5)	6.7
Calcium channel blockers	358 (31.9)	362 (32.3)	0.8
Anti-arrhythmic agent	182 (16.2)	224 (20.0)	9.7
Insulin	301 (26.9)	262 (23.4)	8.0
Alpha-glucosidase inhibitors	14 (1.2)	2 (0.2)	12.7
GLP-1 receptor agonist	6 (0.5)	4 (0.5)	2.7
Meglitinides	123 (11.0)	142 (12.7)	5.3
Metformin	949 (84.7)	937 (83.6)	2.9
Sulfonylureas	451 (40.2)	465 (41.5)	2.5
Thiazolidinediones	85 (7.6)	67 (6.0)	6.4
Health resource use in prior year			
Hospitalizations			
0	892 (70.8)	817 (72.9)	16.2
1–2	201 (17.9)	236 (21.1)	7.9
3+	26 (2.3)	68 (6.1)	18.8
Emergency department visits			
0	50 (4.5)	50 (4.5)	0.0
1–2	561 (50.0)	537 (47.9)	4.3
3+	510 (45.5)	534 (47.6)	4.3

AF, atrial fibrillation; DM, diabetes mellitus; DPP4i, dipeptidyl peptidase-4 inhibitor; GLP-1, glucagon-like peptide-1; SD, standard deviation; SGLT2i, sodium–glucose cotransporter-2 inhibitor; TIA, transient ischaemic attack.

However, compared with the DPP4i group, patients in the SGLT2i group were younger (64.8 vs. 68.0 years). Additionally, the SGLT2i group had fewer patients with a history of HF (35.1% SGLT2i vs. 44.1% DPP4i), peripheral artery disease (3.7 vs. 6.9%), and ischaemic stroke/TIA (18.2 vs. 23.4%).

### Primary outcome

**Figure 3:**
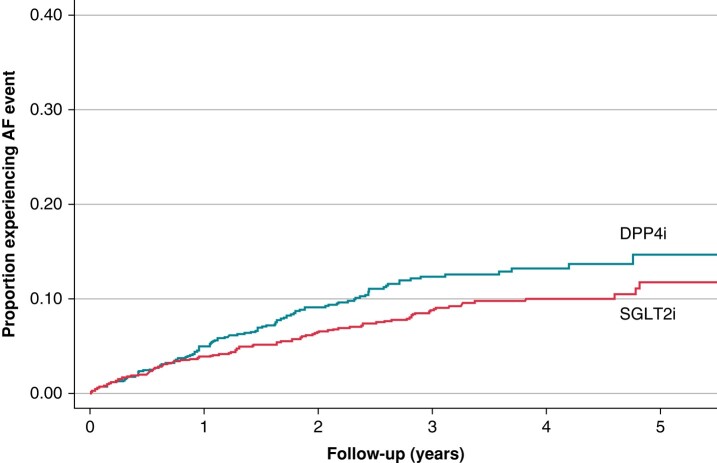
The cumulative incidence of AF events (SGLT2i vs. DPP4i). Cumulative incidence functions for AF events (composite of the first occurrence of an AF-related hospitalization, AF-related emergency department visit, synchronized electrical cardioversion, or catheter ablation). AF, atrial fibrillation; DPP4i, dipeptidyl peptidase-4 inhibitor; SGLT2i, sodium–glucose cotransporter-2 inhibitor.

Over a median follow-up of 3.0 years, 9.3% (*n* = 209) of the matched cohort experienced the primary outcome of an AF event or clinically relevant AF-related medical resource encounter (*Figure 3*). Compared with the DPP4i group, there was a significantly lower risk of experiencing an AF event among patients in the SGLT2i group (adjusted HR 0.73, 95% CI 0.55–0.96; *P* = 0.03; *Table 2*).

**Table 2:** Primary and secondary outcomes by treatment group (propensity score–matched SGLT2i vs. DPP4i users)

Outcomes	SGLT2i *n* = 1121	DPP4i *n* = 1121	Unadjusted HR (95% CI)	*P*-value	Adjusted HR (95% CI)^a^	*P*-value
Primary outcome^b^	8.7 (97)	10.0 (112)	0.73 (0.56–0.96)	0.02	0.73 (0.55–0.96)	0.03
Secondary outcomes						
All-cause mortality	7.2 (81)	33.4 (374)	0.18 (0.14–0.23)	<0.01	0.22 (0.16–0.28)	<0.01
Heart failure hospitalization	6.6 (74)	12.4 (139)	0.42 (0.32–0.56)	<0.01	0.53 (0.40–0.71)	<0.01
All cause hospitalization	43.4 (486)	57.8 (648)	0.58 (0.52–0.66)	<0.01	0.65 (0.58–0.74)	<0.01
Ischaemic stroke/TIA	3.9 (44)	5.1 (57)	0.63 (0.43–0.94)	0.02	0.71 (0.48–1.08)	0.11

AF, atrial fibrillation; CI, confidence interval; DPP4i, dipeptidyl peptidase-4 inhibitor; HR, hazard ratio; SGLT2i, sodium–glucose cotransporter-2 inhibitor; TIA, transient ischaemic attack.

^a^The model includes age, socioeconomic status (material and social Pampalon), heart failure, stroke/TIA, peripheral arterial disease, alpha-glucosidase inhibitor use, and number of hospitalizations in a prior year.

^b^Primary outcome is an ‘AF event’ defined as the composite of first AF-related hospitalization, AF-related emergency department visit, synchronized cardioversion, or catheter ablation.

In the competing risks model, a similar relationship between the primary outcome and the treatment group was observed (see Supplementary material online, *Table S2*). That is, SGLT2i decreased the cumulative incidence of the primary outcome of AF events compared with DPP4i, although this relationship was not statistically significant.

In the recurrent events analysis, the SGLT2i had fewer recurrent AF events compared with the DPP4i group (Andersen–Gill model–adjusted HR 0.61, 95% CI 0.50–0.74; *P* < 0.001), mainly driven by a reduction in AF-related emergency department visits (adjusted HR 0.61, 95% CI 0.49–0.78; *P* < 0.001) and synchronized electrical cardioversions (adjusted HR 0.55, 95% CI 0.40–0.76; *P* < 0.001). This relationship between the treatment group and recurrent events was consistent using the Prentice–William–Peterson total time and frailty models (see Supplementary material online, *Table S3*).

### Secondary outcomes

**Figure 4:**
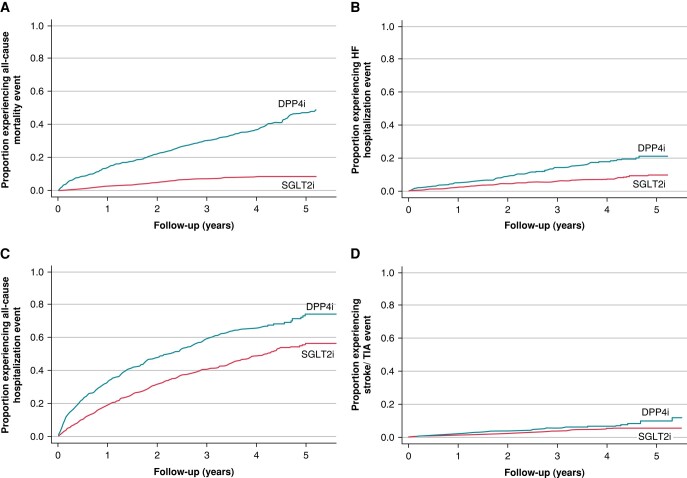
The umulative incidence of secondary outcomes (SGLT2i vs. DPP4i). Cumulative incidence functions for (*A*) all-cause mortality, (*B*) heart failure hospitalization, (*C*) all-cause hospitalization, and (*D*) ischaemic stroke/TIA. DPP4i, dipeptidyl peptidase-4 inhibitor; HF, heart failure; SGLT2i, sodium–glucose cotransporter-2 inhibitor; TIA, transient ischaemic attack.

There were 455 (20.2%) deaths and 101 (4.5%) ischaemic strokes or TIAs observed among the matched cohort. There were a total of 1134 hospitalizations during the study follow-up period, of which 213 were primarily due to HF (*Figure 4*).

Compared with the DPP4i group, patients in the SGLT2i group had a significantly lower risk of all-cause mortality (adjusted HR 0.22, 95% CI 0.16–0.28; *P* < 0.001), all-cause hospitalization (adjusted HR 0.65, 95% CI 0.58–0.74; *P* < 0.001), and HF hospitalization (adjusted HR 0.53, 95% CI 0.40–0.71; *P* < 0.001). There was no difference in ischaemic stroke/TIA between the SGLT2i and DPP4i groups (adjusted HR 0.71, 95% CI 0.48–1.08; *P* = 0.11; *Table 2*). Similar relationships were observed in the competing risk models (see Supplementary material online, *Table S2*). That is, SGLT2i significantly decreased the cumulative incidence of all-cause hospitalization and HF hospitalization compared with DPP4i. Sodium–glucose cotransporter 2 inhibitor use had no significant effect on the subdistribution hazard function of ischaemic stroke/TIA.

### Subgroup analysis

**Figure 5:**
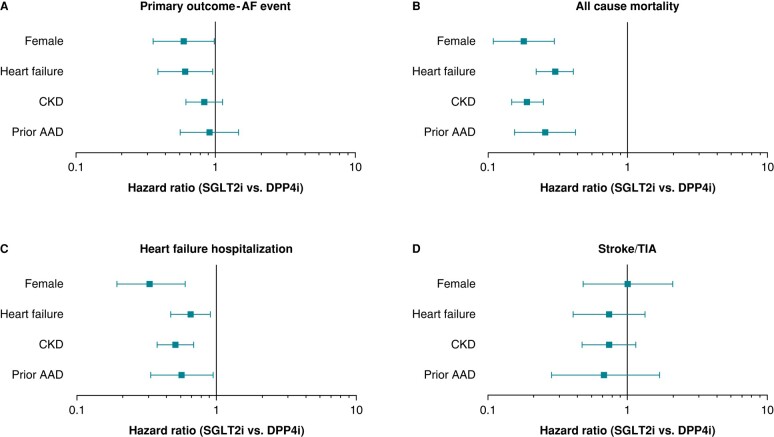
A forest plot summarizing hazard ratios (SGLT2i vs. DPP4i) of study outcomes by subgroup. Subgroup analysis (female, HF, CKD, and prior AAD) for (*A*) AF events (primary outcome), (*B*) all-cause mortality, (*C*) HF hospitalization, and (*D*) stroke/TIA. AF, atrial fibrillation; AAD, anti-arrhythmic drug; CKD, chronic kidney disease; DPP4i, dipeptidyl peptidase-4 inhibitor; HF, heart failure; SGLT2i, sodium–glucose cotransporter-2 inhibitor; TIA, transient ischaemic attack.

The association between SGLT2i prescription and outcomes was assessed in several subgroups (*Figure 5*). In patients with HF at baseline (*n* = 887), the SGLT2i group had fewer AF events compared with the DPP4i group (adjusted HR 0.61, 95% CI 0.39–0.95; *P* = 0.03). A similar benefit was observed in females (*n* = 581; adjusted HR 0.59, 95% CI 0.36–0.98; *P* = 0.04), and there was no significant difference in the primary outcome between treatment groups among patients on anti-arrhythmic medications at baseline or patients with CKD.

In terms of secondary outcome measures, all subgroups (i.e. HF, CKD, female sex, and baseline anti-arrhythmic medications) prescribed an SGLT2i had a significant reduction in all-cause mortality, all-cause hospitalization, and HF hospitalization compared with patients prescribed a DPP4i (see Supplementary material online, *Table S4*). There were no differences between treatment groups in the rate of TIA/stroke.

### Falsification endpoint analysis

The associated treatment–exposure relationships were neutral for the falsification endpoints of chronic obstructive pulmonary disease and lymphoma, which decreases the likelihood of persistent bias. The null hypothesis of neutral association for rheumatoid arthritis was rejected, indicating possible persistent bias; however, the association was in favour of the DPP4i group, which was associated with the lower incidence of rheumatoid arthritis (see Supplementary material online, *Table S5*).

## Discussion

In this population-based analysis of 2242 propensity score–matched patients with pre-existent AF and DM, SGLT2i therapy was associated with a decreased risk of AF-related events and medical resource utilization compared with DPP4i matched controls over a 3-year median follow-up period. Specifically, the risk of recurrent AF events was decreased by approximately one-third among those prescribed an SGLT2i. With regard to secondary study endpoints, SGLT2i conferred a reduced risk of all-cause mortality, HF hospitalization, and all-cause hospitalization. However, there was no difference in stroke/TIA events between the SGLT2i and DPP4i matched groups. Finally, the benefits of SGLT2i were observed across important patient subgroups including patients who were female, had HF, had CKD, or were on anti-arrhythmic drugs at baseline.

The results from our primary analysis are consistent with prior studies^29,30^ that have demonstrated the non-glycaemic effects of SGLT2i in patients with DM, HF, cardiovascular disease, or CKD, such as the CVD-REAL 2, which was a multi-national cohort comparing cardiovascular outcomes between SGLT2i with an active comparator of DPP4i.^31^ Our results are diverse from CVD-REAL 2, which showed a significant reduction in stroke associated with SGLT2i. However, the propensity score matching was conducted in each country separately and differed based on available covariates. Of note, in a meta-analysis of 6 landmark SGLT2i trials comprising 46 969 study participants with DM, SGLT2i did not reduce the risk of ischaemic stroke.^2^

With regard to potential benefits of SGLT2i on AF, prior studies have evaluated the onset of new AF in patients without prior history.^4,32^ For example, in a review of the US Food and Drug Administration adverse events database, AF was less frequently reported in patients on SGLT2i (4.8 vs 8.7/1000; *P* < 0.001) compared with other diabetes medications.^32^ However, there is a relative paucity of literature exploring the impact of SGLT2i on arrhythmia outcomes among patients with pre-existing AF. Recently, Kishima *et al.*^33^ reported the outcomes of 80 patients with paroxysmal or persistent AF and DM undergoing catheter ablation. Patients were randomized to an SGLT2i (tofogliflozin 20 mg daily) or DPP4i (anagliptin 200 mg daily) and followed for 12 months post-ablation for AF recurrence. Recurrent AF was higher in the DPP4i group compared with the SGLT2i group (47 vs. 34%; *P* = 0.04). Our study builds on these findings by demonstrating an association between SGLT2i prescription and reduction in AF events in a broad population of patients with AF and DM. Our findings suggest that SGLT2i may decrease the burden of clinically relevant and recurrent AF events that necessitate healthcare encounters, such as hospitalizations, emergency department visits, catheter ablation, or electrical cardioversion.

Several postulated mechanisms may explain our study findings including potential effects of SGLT2i on electrical and structural atrial remodelling.^34^ In patients with DM, there is up-regulation of the sodium–hydrogen exchanger 1 (NHE1) leading to an increase in intra-cellular sodium, which subsequently increases calcium levels in the sarcoplasmic reticulum due to higher activity of the Na^+^/Ca^2+^ exchanger.^35^ Pro-arrhythmic imbalances in calcium homeostasis may be mitigated by SGLT2i, which suppresses sodium–hydrogen exchange, promotes natriuresis, and decreases cardiac intra-cellular Na^+^ and Ca^2+^.^36,37^ For example, a prior study has shown that human atrial cardiomyocyte NHE1 expression is inhibited by empagliflozin in tissue derived from patients with HF and AF.^38^ These effects have been linked to reduced adverse cardiac remodelling, hypertrophy, and decreased risk of arrhythmias.^39^ Furthermore, SGLT2is have been linked to decreases in epicardial fat, which has been associated with increased AF risk. Sodium–glucose cotransporter 2 inhibitor may also counteract oxidative stress and slow myocardial fibrosis.^39^ Kang *et al.*^40^ found that cardiac myofibroblasts exposed to empagliflozin were smaller in size, had attenuated extracellular matrix remodelling, and had suppression of pro-fibrotic gene markers. Finally, in a subanalysis of the EMPA haemodynamics study, 44 patients with DM were randomized to empagliflozin or placebo and followed with serial transthoracic echocardiography. Empagliflozin improved left atrial function after 3 months of treatment as measured by the left atrial strain compared with placebo.^41^

In summary, the current study contributes to the existing literature and suggests that SGLT2i may reduce the frequency and recurrence of clinically significant AF events necessitating a healthcare encounter. Our findings should be considered exploratory until they can be confirmed by well-powered randomized clinical trials. Several trials are currently planned, but not currently enrolling including the BEYOND trial (Clinical BEnefit of sodium–glucose cotransporter-2 inhibitors in rhYthm cONtrol of atrial fibrillation in patients with diabetes mellitus; NCT05029115) and the EMPA-AF trial (Empagliflozin and Atrial Fibrillation Treatment; NCT04583813). The BEYOND trial aims to enrol 716 patients with AF and DM and randomize participants to an SGLT2i or non-SGLT2i anti-hyperglycaemic agent.^42^ The primary outcome is the recurrence of AF at 12 months. The EMPA-AF trial will enroll 400 patients with DM or body mass index >25 kg/m^2^ and who also have HF and AF. Patients will be randomized to empagliflozin or placebo and followed for 24 months for AF arrhythmic burden.

### Study limitations

Our findings need to be interpreted in the context of several study limitations. Given the non-randomized, retrospective design of the study, we were unable to exclude bias from unmeasured confounders that may influence the association between SGLT2i and study endpoints [such as symptom burden, baseline AF arrhythmic burden, AF subtype (paroxysmal, persistent), or imaging markers of AF recurrence such as left atrial size or left ventricular ejection fraction]. To mitigate bias from measured confounders as best as possible given the observational study design, we used time-conditional propensity score matching and double robust estimation to create comparator cohorts balanced over a large number of measurable variables. Additionally, we compared the SGLT2i cohort with an active comparator (i.e. DPP4i) to reduce the potential for immortal time bias. Nonetheless, we noted a greater-than-expected reduction in mortality associated with the SGLT2i group than previously reported,^43^ suggesting the presence of residual confounders. Second, we did not specifically assess for differences in arrhythmia outcomes by individual SGLT2i (canagliflozin, dapagliflozin, or empagliflozin). Although SGLT2i benefit has been consistently observed in other cardiovascular outcomes across this drug class, we acknowledge the possibility that arrhythmic benefit may not be a medication class effect. Third, our study cohort (that included patients with filled SGLT2i prescriptions) represented a small proportion of the eligible patients for SGLT2is, which may reflect a clinical practice gap and delayed adoption of novel therapies. In this context, our study findings may be generalizable only to patients with similar baseline characteristics; for instance, our cohort was relatively young (mean age 64 years) compared with other reported AF cohorts.^44^ Fourth, the primary study outcome of an AF event relied on administrative claims rather than the traditional definition of >30 s of atrial tachyarrhythmia or AF recurrence. While the current study outcome would not be sensitive to detect all episodes of AF (i.e. short duration or minimally symptomatic), AFs defined through healthcare encounters are clinically meaningful as they represent episodes requiring medical attention due to severity of symptoms or associated clinical decompensation. Our administrative definition of an AF event is consistent with that of prior studies.^45–47^ Furthermore, we would not expect differential detection or diagnostic bias between treatment groups. However, we may have underestimated the potential benefits of SGLT2is on AF burden reduction if continuous electrocardiogram monitoring was used. Fewer AF events may have led to an underpowered analysis, which may account for the lack of statistical significance in the competing risks model evaluating the association between SGLT2i and primary endpoint. Lastly, we noted that overall anticoagulation use was relatively low in our cohort, which may have influenced the rate of stroke during the follow-up period. However, anticoagulation use was similar between the SGLT2i and the DPP4i groups, and the overall low rate of anticoagulation would not be expected to influence the study findings that there may be a benefit of SGLT2i on stroke/TIA.

## Conclusions

Among patients with concomitant DM and AF, the prescription of SGLT2i was associated with fewer AF events, lower risk of all-cause mortality, and fewer HF-related hospitalizations compared with DPP4i. While these results are consistent with the emerging data on the effects of SGLT2i on AF, future well-powered clinical trials are required to confirm these associations, given a possible residual confounding.

## Supplementary material

Supplementary material is available at *Europace* online.

## Supplementary Material

euae054_Supplementary_Data

## Data Availability

The data underlying this article will be shared on reasonable request to the corresponding author.
