# Supplementary material for: Association between sodium–glucose cotransporter-2 inhibitors and arrhythmic outcomes in patients with diabetes and pre-existing atrial fibrillation
Source: Europace. 2024 Mar 14;26(3):euae054. doi: 10.1093/europace/euae054 (PMC10939462; doi:10.1093/europace/euae054)
Supplement: euae054_Supplementary_Data [file euae054_supplementary_data.docx]

**Association between SGLT2 inhibitors and Arrhythmic Outcomes in Patients with Diabetes and Pre-Existing Atrial Fibrillation**

**SUPPLEMENTAL MATERIALS**

Supplemental Table 1. Administrative Codes Used to Define Primary and Secondary Outcomes

Supplemental Table 2. Association Between Outcomes and Treatment Group with Competing Risk of Death

Supplemental Table 3: Recurrent Events Analysis

Supplemental Table 4. Subgroup Analyses

Supplemental Table 5: Falsification Endpoint Analysis

**Supplemental Table 1. Administrative Codes Used to Define Primary and Secondary Outcomes**

| Outcome | Description | Data source | ICD-9-CM | ICD-10-CA | Health Service Codes |
| --- | --- | --- | --- | --- | --- |
| Hospitalization for Atrial fibrillation and flutter | (most responsible) First hospitalization or emergency department visit for AF/AFL | Alberta Health (hospitalization) | 427.31  427.32 | I48.x | n/a |
| Emergency Department Visit for AF/AFL | Emergency department visit for AF/AFL | Alberta Health (ACCS – emergency department) | 427.31  427.32 | I48.x | n/a |
| Electrical Cardioversion | Electrical cardioversion for AF/AFL | Physician Claims | n/a | n/a | 13.72A  49.98Y |
| Catheter Ablation | Catheter Ablation for AF/AFL | Physician Claims | n/a | n/a | 49.98AB  49.98AC |
| Ischemic stroke  (definition by: Kokotailo et al. Stroke 2005;36:1776-81) | (most responsible) First hospitalization or emergency department visit for ischemic stroke | Alberta Health (hospitalization); Alberta Health (ACCS – emergency department) | 362.3  433.x  434.x  436 | H34.1  H34.2  I63.x  I64.x | n/a |
| TIA  (definition by: Kokotailo et al. Stroke 2005;36:1776-81) | (most responsible) First hospitalization or emergency department visit for ischemic stroke | Alberta Health (hospitalization); Alberta Health (ACCS – emergency department) | 435.x | G45.x (excluding G45.4: transient global amnesia) | n/a |
| Heart Failure Hospitalization  (definition by:  Quan et al. Med Care 2005; 43:1130-9.  Birchman-Deych et al. Med Care 2005;43:480-5) | (most responsible) First hospitalization | Alberta Health (hospitalization) | 398.91, 402.01, 402.11, 402.91, 404.01, 404.03, 404.11, 404.13, 404.91, 404.93, 428.* | I09.81, I11.0*, I13.0*, I13.2*, I50.* | n/a |

**Supplemental Table 2. Association Between Outcomes and Treatment Group with Competing Risk of Death**

| **Outcomes** | **Unadjusted HR***  **(95% CI)** | **p-value** | **Adjusted HR**  **(95% CI)** | **p-value** |
| --- | --- | --- | --- | --- |
| Primary Outcome** | 0.85 (0.65 – 1.12) | 0.25 | 0.83 (0.63 – 1.10) | 0.19 |
| Secondary Outcomes |  |  |  |  |
| Heart Failure Hospitalization | 0.50 (0.38 – 0.67) | <0.01 | 0.66 (0.49 – 0.89) | <0.01 |
| All Cause Hospitalization | 0.62 (0.55 – 0.69) | <0.01 | 0.71 (0.63 – 0.80) | <0.01 |
| Ischemic Stroke / TIA | 0.76 (0.51 – 1.12) | 0.17 | 0.91 (0.60 – 1.39) | 0.67 |

* Hazard ratio is comparing SGLT2i versus DPP4i

**Primary outcome is an “AF Event” defined as composite of first AF-related Hospitalization, AF-related emergency department visit, synchronized cardioversion or catheter ablation.

Abbreviations: AF – atrial fibrillation; CI – confidence interval; DPP4i – Dipeptidyl peptidase-4 inhibitor; SGLT2i – Sodium/glucose cotransporter-2 inhibitor.

**Supplemental Table 3. Recurrent Events Analysis of the Primary Composite Endpoint and Components of the Composite**

|  | **SGLT2i**  **N=1,121** | **DPP4i**  **N=1,121** | **Unadjusted HR (95% CI)** | **p-value** | ^†^**Adjusted HR** **(95% CI)** | **p-value** |
| --- | --- | --- | --- | --- | --- | --- |
| **Composite Outcome*** |  |  |  |  |  |  |
| AG model | 187 | 246 | 0.63 (0.43 – 0.93) | 0.02 | 0.61 (0.50 – 0.74) | <0.01 |
| PWP -TT model | 187 | 246 | 0.74 (0.60 – 0.92) | 0.01 | 0.80 (0.60 – 0.91) | <0.01 |
| Frailty model | 187 | 246 | 0.62(0.45 – 0.87) | 0.01 | 0.64 (0.45 – 0.91) | 0.01 |
| **AF-Related Hospitalizations** |  |  |  |  |  |  |
| AG model | 58 | 60 | 0.81 (0.44 – 1.48) | 0.49 | 0.86 (0.53 – 1.08) | 0.55 |
| PWP -TT model | 58 | 60 | 0.58 (0.38 – 0.88) | 0.01 | 0.62 (0.40 – 0.96) | 0.03 |
| Frailty model | 58 | 60 | 0.78 (0.35 – 1.71) | 0.53 | 0.81 (0.35 – 1.88) | 0.63 |
| **AF-Related Emergency Department Visits** |  |  |  |  |  |  |
| AG model | 128 | 166 | 0.65 (0.42 – 0.99) | 0.05 | 0.61 (0.49 – 0.78) | <0.01 |
| PWP -TT model | 128 | 166 | 0.82 (0.63 – 1.06) | 0.13 | 0.80 (0.62 – 1.30) | 0.08 |
| Frailty model | 128 | 166 | 0.65 (0.45 – 0.95) | 0.03 | 0.64 (0.43 – 0.95) | 0.03 |
| **Synchronized Cardioversion** |  |  |  |  |  |  |
| AG model | 67 | 89 | 0.63 (0.36 – 1.09) | 0.099 | 0.55 (0.40 – 0.76) | <0.01 |
| PWP -TT model | 67 | 89 | 0.71 (0.32 – 1.55) | 0.39 | 0.634 (0.29 – 1.41) | 0.26 |
| Frailty | 67 | 89 | 0.63 (0.38 – 1.05) | 0.07 | 0.57 (0.33 – 0.99) | 0.045 |
| **Catheter Ablation** |  |  |  |  |  |  |
| AG model | 11 | 14 | 0.67 (0.27 – 1.68) | 0.40 | 0.575 (0.26 – 1.29) | 0.18 |
| PWP -TT model | 11 | 14 | 0.71 (0.32 – 1.55) | 0.39 | 0.634 (0.29 – 1.41) | 0.26 |
| Frailty model | 11 | 14 | 0.67 (0.27 – 1.65) | 0.38 | 0.64 (0.24 – 1.69) | 0.37 |

*Composite of AF-related Hospitalizations, AF-related ED Visits, Synchronized Cardioversion or Catheter Ablation.

Abbreviations: AF – atrial fibrillation; AG – Andersen-Gill; CI – confidence interval; DPP4i – Dipeptidyl peptidase-4 inhibitor; PWP-TT – Prentice-William-Peterson Total-Time; SGLT2i – Sodium/glucose cotransporter-2 inhibitor.

**Supplemental Table 4. Subgroup Analyses of Primary and Secondary Outcomes by Treatment Group**

|  | **SGLT2** | **DPP4** | **Unadjusted HR (95% CI)** | **p-value** | ^†^**Adjusted HR** **(95% CI)** | **p-value** |
| --- | --- | --- | --- | --- | --- | --- |
| **Heart Failure** | **N=393** | **N=494** |  |  |  |  |
| Primary Outcome* | 8.4 (33) | 11.1 (55) | 0.61 (0.40 – 0.95) | 0.03 | 0.61 (0.387 – 0.950) | 0.03 |
| Secondary Outcomes |  |  |  |  |  |  |
| All-Cause Mortality | 13.2 (52) | 45.1 (223) | 0.25 (0.18 – 0.34) | <0.01 | 0.302 (0.222 – 0.411) | <0.01 |
| Heart Failure Hospitalization | 15.3 (60) | 20.5 (101) | 0.60 (0.44 – 0.84) | <0.01 | 0.655 (0.471 – 0.911) | 0.01 |
| All Cause Hospitalization | 52.9 (208) | 67.8 (335) | 0.60 (0.50 – 0.71) | <0.01 | 0.679 (0.568 – 0.810) | <0.01 |
| Ischemic Stroke / TIA | 4.8 (19) | 6.5 (32) | 0.62 (0.356 – 1.11) | 0.11 | 0.743 (0.411 – 1.342) | 0.32 |
| **Female Sex** | **N=277** | **N=304** |  |  |  |  |
| Primary Outcome* | 9.8 (27) | 12.2 (37) | 0.63 (0.38 – 1.04) | 0.07 | 0-.592 (0.356 – 0.981) | 0.04 |
| Secondary Outcomes |  |  |  |  |  |  |
| All-Cause Mortality | 6.1 (17) | 33.9 (103) | 0.15 (0.089 – 0.25) | <0.01 | 0.181 (0.107 – 0.307) | <0.01 |
| Heart Failure Hospitalization | 6.5 (18) | 15.5 (47) | 0.31 (0.18 – 0.54) | <0.01 | 0.334 (0.194 – 0.597) | <0.01 |
| All Cause Hospitalization | 48.4 (134) | 61.8 (188) | 0.57 (0.46 – 0.72) | <0.01 | 0.621 (0.493 – 0.782) | <0.01 |
| Ischemic Stroke / TIA | 5.1 (14) | 5.6 (17) | 0.74 (0.37 – 1.51) | 0.41 | 1.013 (0.483 – 2.126) | 0.97 |
| **Prior Antiarrhythmic Use** | **N=182** | **N=244** |  |  |  |  |
| Primary Outcome* | 20.9 (38) | 15.6 (35) | 1.06 (0.67 – 1.68) | 0.80 | 0.904 (0.559 – 1.461) | 0.68 |
| Secondary Outcomes |  |  |  |  |  |  |
| All-Cause Mortality | 11.0 (20) | 42.9 (96) | 0.20 (0.12 – 0.32) | <0.01 | 0.257 (0.155 – 0.426) | <0.01 |
| Heart Failure Hospitalization | 14.3 (26) | 21.0 (47) | 0.49 (0.30 – 0.80) | <0.01 | 0.568 (0.338 – 0.955) | 0.03 |
| All Cause Hospitalization | 52.2 (95) | 68.3 (152) | 0.55 (0.43 – 0.72) | <0.01 | 0.626 (0.479 – 0.821) | <0.01 |
| Ischemic Stroke / TIA | 5.0 (9) | 6.7 (15) | 0.59 (0.26 – 1.36) | 0.22 | 0.678 (0.286 – 1.715) | 0.41 |
| **Chronic Kidney Disease** | **N=912** | **N=941** |  |  |  |  |
| Primary Outcome* | 9.3 (85) | 9.4 (88) | 0.81 (0.60 – 1.09) | 0.16 | 0.831 (0.614 – 1.126) | 0.23 |
| Secondary Outcomes |  |  |  |  |  |  |
| All-Cause Mortality | 7.6 (69) | 39.5 (372) | 0.16 (0.12 – 0.20) | <0.01 | 0.192 (0.148 – 0.250) | <0.01 |
| Heart Failure Hospitalization | 7.6 (69) | 14.3 (135) | 0.40 (0.30 – 0.54) | <0.01 | 0.507 (0.377 – 0.684) | <0.01 |
| All Cause Hospitalization | 47.0 (429) | 61.6 (580) | 0.57 (0.50 – 0.65) | <0.01 | 0.646 (0.568 – 0.735) | <0.01 |
| Ischemic Stroke / TIA | 4.2 (38) | 5.1 (48) | 0.65 (0.42 – 0.92) | 0.05 | 0.743 (0.478 – 1.154) | 0.19 |

* Primary Outcome is an “AF Event” defined as composite of first AF-related Hospitalization, AF-related emergency department visit, synchronized cardioversion, or catheter ablation.

^†^ Model includes age, socioeconomic status (Material and Social Pampalon), heart failure, stroke/TIA, peripheral arterial disease, alpha glucosidase inhibitor use, and number of hospitalizations in prior year. Note: For the heart failure subgroup, heart failure was not included in the model as a covariate.

Abbreviations: AF – atrial fibrillation; CI – confidence interval; DPP4i – Dipeptidyl peptidase-4 inhibitor; SGLT2i – Sodium/glucose cotransporter-2 inhibitor.

**Supplemental Table 5. Falsification Endpoint Analysis**

|  | **SGLT2i** | **DPP4i** | **Unadjusted HR (95% CI)** | **p-value** | ^†^**Adjusted HR** **(95% CI)** | **p-value** |
| --- | --- | --- | --- | --- | --- | --- |
| COPD | 2.9 (32) | 4.6 (51) | 0.71 (0.45 – 1.12) | 0.14 | 0.69 (0.44 – 1.10) | 0.12 |
| Lymphoma | 0.2 (2) | 1.2 (13) | 0.18 (0.04 – 0.80) | 0.03 | 0.24 (0.05 – 1.08) | 0.06 |
| Rheumatoid Arthritis | 1.0 (11) | 0.2 (2) | 5.49 (1.21 – 24.8) | 0.03 | 7.43 (1.61 – 34.4) | 0.01 |

^†^ Adjusted Cox proportional hazard models with competing risk of death includes age, socioeconomic status (Material and Social Pampalon), heart failure, stroke/TIA, peripheral arterial disease, alpha glucosidase inhibitor use, and number of hospitalizations in prior year.
